# An Innovative Database for Epidemiological Field Studies of Neglected Tropical Diseases

**DOI:** 10.1371/journal.pntd.0000413

**Published:** 2009-05-26

**Authors:** Darren J. Gray, Simon J. Forsyth, Robert S. Li, Donald P. McManus, YueSheng Li, Honggen Chen, Feng Zheng, Gail M. Williams

**Affiliations:** 1 Molecular Parasitology Laboratory, Queensland Institute of Medical Research, Brisbane, Queensland, Australia; 2 The School of Population Health, The University of Queensland, Brisbane, Queensland, Australia; 3 Hunan Institute of Parasitic Diseases, WHO Collaborating Centre for Research and Control on Schistosomiasis in Lake Region, Yueyang, People's Republic of China; 4 Jiangxi Provincial Institute of Parasitic Diseases, Nanchang, People's Republic of China; 5 Institute of Parasitic Diseases, Chinese Centre for Disease Control and Prevention, Shanghai, People's Republic of China; George Washington University, United States of America

## Introduction

The neglected tropical diseases (NTDs) are of major public health importance, accounting for 56.6 million disability-adjusted life years (DALYs), which places them sixth out of the ten leading causes of life years lost to disability and premature death [1]. These diseases are prominent in the developing world where there is low income, poor hygiene, and inadequate sanitation [1],[2]. Recent targeting of these diseases for large-scale control programs by the World Health Organization [3] is likely to increase the number of epidemiological field studies requiring valid and reliable data, in order to determine the most appropriate strategies for control.

In order to ensure a control strategy is effective and appropriate, the data need to be of a high standard, and as a result, epidemiological field studies require a rigorous and systematic approach to data management. Recent publications by Ali et al. [4] and Roberts et al. [5] stress that the importance of data management is often underestimated in such studies, with greater emphasis instead placed on the study design, data collection, and data analysis [4],[5]. This can result in an ad hoc approach to data management that ultimately affects the reliability and validity of the data collected and increases the workload involved in data cleaning. There are additional difficulties in developing countries in the collection, entry, management, and analysis of high-quality data, mainly due to limited infrastructure and capacity [4]–[7], which can exacerbate the problems associated with ensuring effective and reliable data management.

We undertook an epidemiological study of the transmission dynamics of *Schistosoma japonicum* in China [8] that necessitated a rigorous approach to the collection and management of an extensive dataset. Some technical and conceptual constraints were encountered as the data management protocols in place were designed for the monitoring and control of schistosomiasis, rather than for the evaluation of a complex epidemiological study, requiring expertise in the principles and practice of data management. Language barriers provided additional challenges in implementing an efficient data management system.

Accordingly, we present details of the innovative database we developed, which allowed us to produce data that were protected against data entry errors and therefore more likely to be of high quality and reliability. Furthermore, it also provided us with evidence of protection. This database can also serve as a template for other epidemiological studies of NTDs in the future.

## The Database System

The database was developed to process data collected during a field-based intervention trial to determine the importance of bovines in *S. japonicum* transmission in southern China [8]. The study involved four matched pairs of villages around Dongting lake (Hunan Province) and Poyang lake (Jiangxi Province), with a village within each pair randomly selected as intervention (human and bovine praziquantel treatment) or control (human praziquantel treatment only). The primary end point of the trial was human infection incidence. The trial design, being complex, required a database with numerous data entry forms, corresponding tables, and specified inter-table relationships. The database incorporated a Microsoft Access framework with Visual Basic (VBA) modules and SQL scripts. To allow for gold standard double entry [9], each village had two database files, one for each of the first and second data entries.

Microsoft Access was used because it is widely supported, has functionality for quality assurance, and provides greater flexibility for questionnaire design compared to other systems such as Epi Info and EpiData. Although EpiData and Epi Info have more extensive analyses available, the Microsoft Access framework is focused on correct and validated data entry with an analysis component added to: (a) provide immediate feedback to the researcher in the field; and (b) provide another layer of data verification. In practice, more complex analyses should be reserved for more powerful statistical software such as SAS (SAS Institute; http://www.sas.com/), which can directly use the Microsoft Access data.

**Figure 1:**
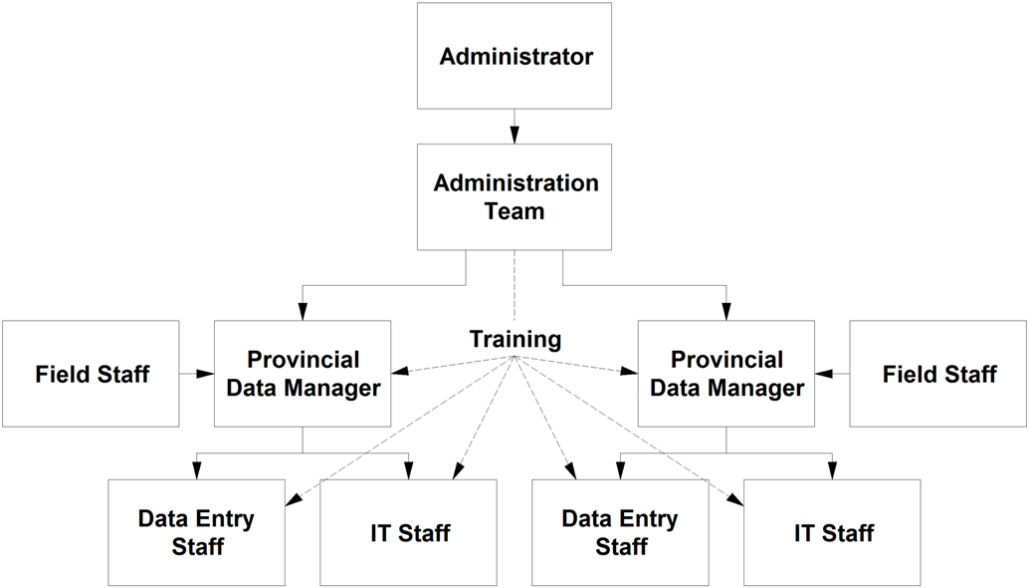
Data management organizational framework.

Prior to the implementation of the database, an organizational framework (Figure 1) was established to provide the infrastructure from which the database system could be built [4]. Imposition of a file management structure creates standardization, a key factor in producing quality data, which is particularly important when there are multiple sites for data entry.

The database was developed with innovative functions, which included: a user-friendly bilingual Chinese/English interface, an automated codebook generator, an automated real-time internal double-entry check, an external final error check with audit trail analysis, and built-in statistical functionality (Box 1).

**Box 1. Advantages and Disadvantages of the Database**

**Advantages:**

- Efficient data entry and error checking
- Audit trail for quality control
- Quality control metrics to detect sources of error, bias, data entry speed, and accuracy
- Built-in statistical analysis functionality
- Bilingual Chinese/English interface
- Automated codebook generation

**Disadvantages:**

- Potential bias towards either first or second entry if data is incorrectly entered
- Internal double-entry check is unable to correct ID variable errors
- Restricted Microsoft Windows operating system

**Figure 2:**
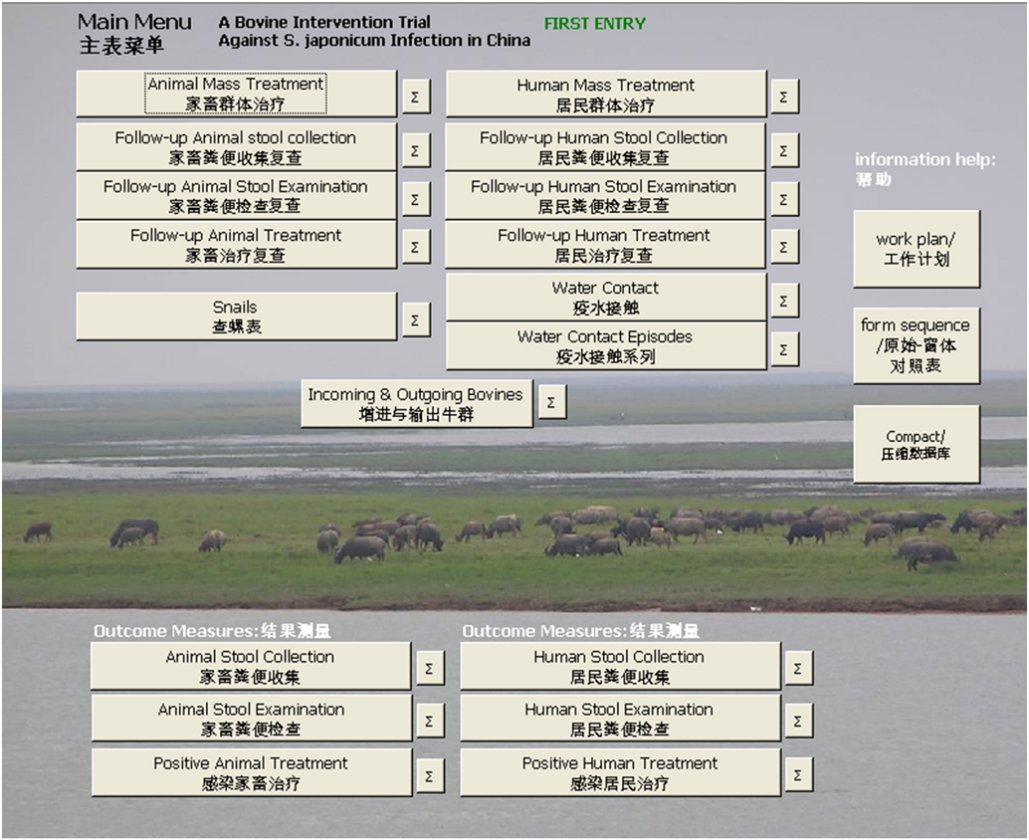
Screenshot of database main menu.

The user-friendly bilingual Chinese/English interface allows cross-cultural users to easily navigate through the system and perform the data entry, which therefore only requires a basic level of computing skills (Figure 2).

The automated real-time double-entry check is a key component of the database. During the second data entry, an automatic comparison is made between the value just entered for a particular field with the value entered for that field in the first entry. Users are notified of any discrepancies between the two datasets and are prompted to select the correct value or defer to follow-up on the discrepancy. The advantage of this feature is that the user can immediately refer to the original paper form in order to confirm the correct value for a particular field. This process is logged for audit trail analysis and quality control.

This approach differs from the traditional method of double entry in that error checking is performed live during the second data entry, thereby reducing the number of passes through the data and the time involved in sorting through the paper forms. The traditional method requires the entry of both datasets to be completed before the error checking process can begin. Data entry is not considered to be complete until both datasets are free from errors, which can take multiple passes through the data.

The final external error check is an external script designed as a fail-safe error checking process and is somewhat like the traditional double-entry record checking and reporting procedure. Differences between the first and second entries are identified, which data entry staff can subsequently correct. The data entry is considered complete when there are no differences between the two files. The audit trail error analysis performed by this script is a form of data entry quality control as it evaluates the performance of the data entry staff and the internal double-entry check function. Anomalies in the results or a bias towards either the first or second entry prompt further investigation as to the source of the discrepancy.

The ready-made statistical analysis feature—validated within SAS—is a set of pre-programmed (VBA and SQL) statistical equations, relevant to (a) all studies (frequencies and cross-tables), and (b) the ongoing intervention trial (prevalence), although this can be tailored to any study. This feature enables rapid simple statistics to be performed following the completion of the data entry. This serves as another error checking mechanism and also obviates the need for the user to have complex statistical skills.

While other pre-packaged database systems (e.g., EpiData) may have some similar functions to the database framework described here, this database system is able to deal with more complex form designs, and linkages for live double-entry comparisons. Furthermore, the quality control functionality is superior to other packages and the output generated demonstrates the validity of the data and provides users with quality assurance. Therefore, it is more advantageous for users when undertaking epidemiological field studies in environments with limited infrastructure and capacity for data management.

The coding for the VBA modules and SQL scripts along with the associated instruction manual can be accessed at http://hisdu.sph.uq.edu.au/msadb/.

## Future Applications

Epidemiological field studies of other NTDs in developing countries with limited infrastructure and technical capabilities for data management will likely face similar challenges to those we experienced. The concept and principles of the database we describe can serve as a template for the data management of these studies. Furthermore, the implementation of the database and associated educational workshops can contribute to capacity building and technology transfer when working in an environment with limited resources. We found that staff training was important in the implementation and subsequent use of the database system. Additional data management and statistical analysis workshops provided staff with a good knowledge base in the principles and practice of data management, as well as the statistical principles of study design and descriptive statistics.

## Conclusions

Researchers using this database framework are more likely to produce good-quality, reliable data via the self-validating functionality, quality control reporting, maximization of the rate of data transfer from the paper to electronic form, and reduced time in data cleaning procedures.

## References

[1] Hotez PJ, Molyneux DH, Fenwick A, Kumaresan J, Sachs SE (2007). Control of neglected tropical diseases.. N Eng J Med.

[2] [No authors listed] (2007). Remembering the neglected tropical diseases.. Lancet.

[3] World Health Organization (2006). Neglected tropical diseases: Hidden successes, emerging opportunities.. http://www.popline.org/docs/1729/312303.html.

[4] Ali M, Park JK, von Seidlein L, Acosta CJ, Deen JL (2006). Organizational aspects and implementation of data systems in large-scale epidemiological studies in less developed countries.. BMC Pub Health.

[5] Roberts RJ, Musick BS, Olley B, Hall KS, Hendrie HC (2000). Data management in a longitudinal cross-cultural study.. Stat Med.

[6] Kimbrough Pradhan E, Katz J, LeClerq SC, West KP (1994). Data management for large community trials in Nepal.. Control Clinic Trial.

[7] Ma J, Otten M, Kamadjeu R, Mir R, Rosencrans L (2008). New frontiers for health information systems using Epi Info in developing countries: Structured application framework for Epi Info (SAFE).. Int J Med Inform.

[8] Gray DJ, Williams GM, Li Y, Chen H, Li RS (2007). A cluster-randomised bovine intervention trial against *S. japonicum* in the People's Republic of China: Design and baseline results.. Am J Trop Med Hyg.

[9] Reynolds-Haertle RA, McBride R (1992). Single vs. double entry in CAST.. Control Clinic Trial.

